# HOUSING MODELS TO PREVENT AND INTERVENE IN HOMELESSNESS AMONG LGBTQIA+ OLDER ADULTS

**DOI:** 10.1093/geroni/igae098.1493

**Published:** 2024-12-31

**Authors:** Angela Perone, Leyi Zhou

**Affiliations:** University of California Berkeley, Berkeley, California, United States; University of California Berkeley, Berkeley, California, United States

## Abstract

LGBTQIA+ older adults have heightened risks for homelessness, given structural and systemic barriers and inequities across the life course. Little is known about housing prevention and intervention models for LGBTQ+ older adults at risk of homelessness. This study uses a novel multi-method approach to answer the following community-driven research questions: What housing models aim to prevent or intervene in homelessness among LGBTQIA+ older adults? How do these models respond to the diverse needs of LGBTQIA+ older adults? The research team first conducted a scoping review using Arksey and O’Malley’s five-step framework. In collaboration with a national community partner, the team next identified and reviewed grey literature. The team supplemented review findings with secondary qualitative data from community leaders. Data revealed five core housing models used as prevention and intervention homelessness tools among LGBTQIA+ older adults: affordable housing developments, homeshare spaces, tiny homes, community land trusts, and mobile communities. Affordable housing developments tended to be located in spaces that were more heavily populated by white residents. Transgender and LGBTQIA+ older adults of color led projects that invoked the other four nontraditional housing models. While affordable housing developments for LGBTQIA+ older adults are growing, they may not be meeting the diverse needs of this community. Intersecting experiences of trauma, discrimination, and exclusion (e.g., racism, ableism, sexism, heterosexism, transphobia) may be driving the need and desire among transgender older adults and LGBTQIA+ older adults of color to develop alternative housing models to prevent homelessness.

